# Graphene-enhanced metal oxide gas sensors at room temperature: a review

**DOI:** 10.3762/bjnano.9.264

**Published:** 2018-11-09

**Authors:** Dongjin Sun, Yifan Luo, Marc Debliquy, Chao Zhang

**Affiliations:** 1 College of Mechanical Engineering, Yangzhou University, Yangzhou 225127, China; 2 Department of Materials Science, University of Mons, 7000 Mons, Belgium

**Keywords:** gas sensor, graphene, metal oxide, nitrogen dioxide (NO2), room temperature

## Abstract

Owing to the excellent sensitivity to gases, metal-oxide semiconductors (MOS) are widely used as materials for gas sensing. Usually, MOS gas sensors have some common shortages, such as relatively poor selectivity and high operating temperature. Graphene has drawn much attention as a gas sensing material in recent years because it can even work at room temperature, which reduces power consumption. However, the low sensitivity and long recovery time of the graphene-based sensors limit its further development. The combination of metal-oxide semiconductors and graphene may significantly improve the sensing performance, especially the selectivity and response/recovery rate at room temperature. In this review, we have summarized the latest progress of graphene/metal-oxide gas sensors for the detection of NO_2_, NH_3_, CO and some volatile organic compounds (VOCs) at room temperature. Meanwhile, the sensing performance and sensing mechanism of the sensors are discussed. The improved experimental schemes are raised and the critical research directions of graphene/metal-oxide sensors in the future are proposed.

## Review

### Introduction

Since the discovery by Novoselov and Geim [1], graphene has been widely used in various fields such as photocatalysts, lithium battery electrodes, supercapacitors, gas sensors and electronic devices [2–4] due to its high specific surface area (2630 m^2^/g) and high carrier mobility at room temperature [5]. The electrical properties of graphene are easily affected by the adsorption of gas molecules at room temperature. Thus, graphene has a promising future in the application in gas sensors. Schedin et al. [2] studied the gas sensing performance of graphene for the first time in 2007 and claimed that the adsorption of gas molecules caused a gradual change in graphene resistance via altering the local carrier concentration. After that, a wave of research regarding graphene has been set off.

The mass production of single-layered graphene is difficult. Another problem is that pristine graphene does not have a bandgap, which means it is not suitable for semiconductor gas sensors [6]. Graphene oxide (GO), as a derivative of graphene, is prepared via the oxidation of graphene. Epoxy groups, hydroxy groups and defects are produced at the surface when oxidizing graphene [7–10]. These variations will alter the electronic structure of graphene, thus converting it to a semiconductor. Choi et al. [11] prepared GO room-temperature gas sensors by a modified Hummers method. The group found that the sensitivity and repeatability of the sensor depended on the amount of oxygen functional groups on the surface of GO. Moreover, hydroxy groups were the key to provide GO with semiconducting properties. However, an excessive presence of functional groups will make GO an insulating material [12]. In addition, it is difficult to control the content of oxygen functional groups during the process of oxidation, indicating that GO is not an appropriate gas-sensing material. Therefore, further reduction of GO is necessary and the product after reduction is called reduced graphene oxide (rGO). Some oxygen functional groups remain after the reduction, some defects and vacancies are generated during the reduction, which are beneficial for the gas adsorption [13]. The oxygen functional groups that locate on the surface of rGO lead to an electron transfer from rGO to oxygen functional groups, and holes become the main charge carriers, indicating that rGO acts as a p-type semiconductor [14–16]. Zhang et al. [17] prepared rGO room-temperature gas sensor with porous structure and defects for detecting NO_2_. The sensor showed high sensitivity to NO_2_ at low concentrations. In another work, Hu et al. [18] fabricated an ultra-sensitive rGO gas sensor, which reached a response of 2.4% to 1 ppb NH_3_ with an ultra-fast response time of 1.4 s at room temperature. The sensors based on rGO exhibited a rapid and high response to target gas at room temperature. However, these sensors show a common shortage. Since the binding force between graphene and gas molecules is van der Waals force or even covalent bonds [6], the recovery time is too long, sometimes recovery is not achieved at all [19–20].

Metal-oxide semiconductors (MOS), including tin oxide (SnO_2_), titanium dioxide (TiO_2_), zinc oxide (ZnO), copper oxide (CuO), tungsten oxide (WO_3_), indium oxide (In_2_O_3_), ferric oxide (Fe_2_O_3_) and cobalt oxide (Co_3_O_4_) are important materials for gas sensors [21–28]. These types of materials possess many exceptional advantages, such as high sensitivity, rapid response/recovery times and low cost. Until now, there is no unified definition of the mechanism of MOS gas sensors. The most widely accepted oxygen-adsorption theory is described as follows [29]: oxygen molecules capture electrons from semiconductors to form oxygen anions when the sensor is exposed to air, and the operating temperature affects the forms of oxygen anions. When the operating temperature is below 147 °C, the oxygen anions are mainly O_2_^−^. With the increase of temperature, O_2_^−^ is transformed into O^−^. When the temperature is above 397 °C, the oxygen anions are converted into O^2−^. The reaction equations are as follows:

[1]
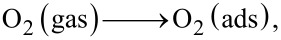


[2]
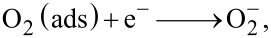


[3]
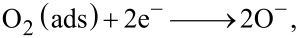


[4]
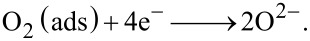


For n-type semiconductors, the electrons will continue to be captured from the surface of the semiconductors so that the width of electron depletion layer increases when exposed to oxidizing gases, increasing the electrical resistance. The gas molecules act as electron donors to the semiconductors when exposed to reducing gases, meaning that the width of electron depletion layer decreases, which decreases the resistance of the sensor. For p-type semiconductors, the width of hole accumulation layer increases by capturing electrons from the surface of semiconductors when exposed to oxidizing gases, causing the resistance to decrease. The electrons are released into the semiconductor to decrease the width of hole accumulation layer when exposed to reducing gases, which increases the resistance of the sensor. The sensing performances of MOS sensors are heavily affected by the working temperature, because the working temperature influences the kinetics, conductivity and electron mobility of MOS [30–31]. Since sufficient thermal energy is required to overcome the potential barrier and achieve the required electron mobility, the operating temperature of MOS sensors is above 200 °C in general. The excessive operating temperature leads to high power consumption and difficulty of integration, which is contrary to our concept of energy conservation and emission reduction. Moreover, sensors working in flammable and explosive environments at a high temperature may cause fire or explosion. Also, operating at high temperature causes sensor instabilities, which lead to incorrect measurements [32–34]. Therefore, current research focuses on reducing the operating temperature of MOS gas sensors.

Modifications of composition and surface, and light illumination of MOS are effective ways to improve their gas-sensing performance. MOS composites with graphene or its derivatives can reduce the operating temperature and yield outstanding sensing performance surpassing that of the single components. The mechanisms through which graphene enhances the sensing performance of MOS sensors will be interpreted in the following sections. Wang et al. [35] reported that a formaldehyde (HCHO) sensor based on SnO_2_–GO composites, fabricated via electrospinning, exhibited a three times higher sensitivity than that of the pure SnO_2_ sensor at 120 °C. The composite sensor was able to detect 500 ppb HCHO. The unique sensing properties of SnO_2_–GO sensor was interpreted by the large specific surface area, the high number of oxygen functional groups and electric regulation effects provided through the addition of GO. The ZnO–rGO sensor reported by Zou et al. [36] showed a sensitivity of 96.4 to 50 ppm ethanol at 260 °C with short response and recovery times. Extensive research on graphene/metal-oxide sensors has been carried out over the recent years [37–43]. It appears clear that the working temperature of graphene/metal-oxide sensors is lower than that of MOS sensors. In some cases, graphene/metal-oxide sensors were even operated at room temperature.

There are many kinds of toxic gases from industrial processes and car emissions around us, such as NO_2_, NH_3_, CO and most volatile organic compounds (VOCs). In general, people should not be exposed to an environment with more than 35 ppm NH_3_ for more than 15 min or an environment with more than 10 ppm CO for more than 10 min. Also, people should not be exposed to workplaces with more than 0.08 ppm formaldehyde for more than 30 min according to World Health Organization [44]. Therefore, there is an urgent need for gas sensors with low cost, outstanding selectivity and sensitivity for detecting these toxic gases [6]. Compared with MOS sensors, graphene/metal-oxide sensors enhance the gas-sensing performance in many aspects, such as sensitivity, response/recovery times and the operating temperature. There are numerous mechanisms for the enhanced gas-sensing performance according to different views of scholars. The most widely accepted mechanisms are: the formation of semiconductor interfaces, a synergetic coupling effect between the two components, and improved morphology and structure due to the introduction of graphene. The following is a review of the recent progress concerning the application of graphene/metal-oxide sensors to discern various toxic gases at room temperature.

### Enhancement by the formation of semiconductor interfaces

**Figure 1:**
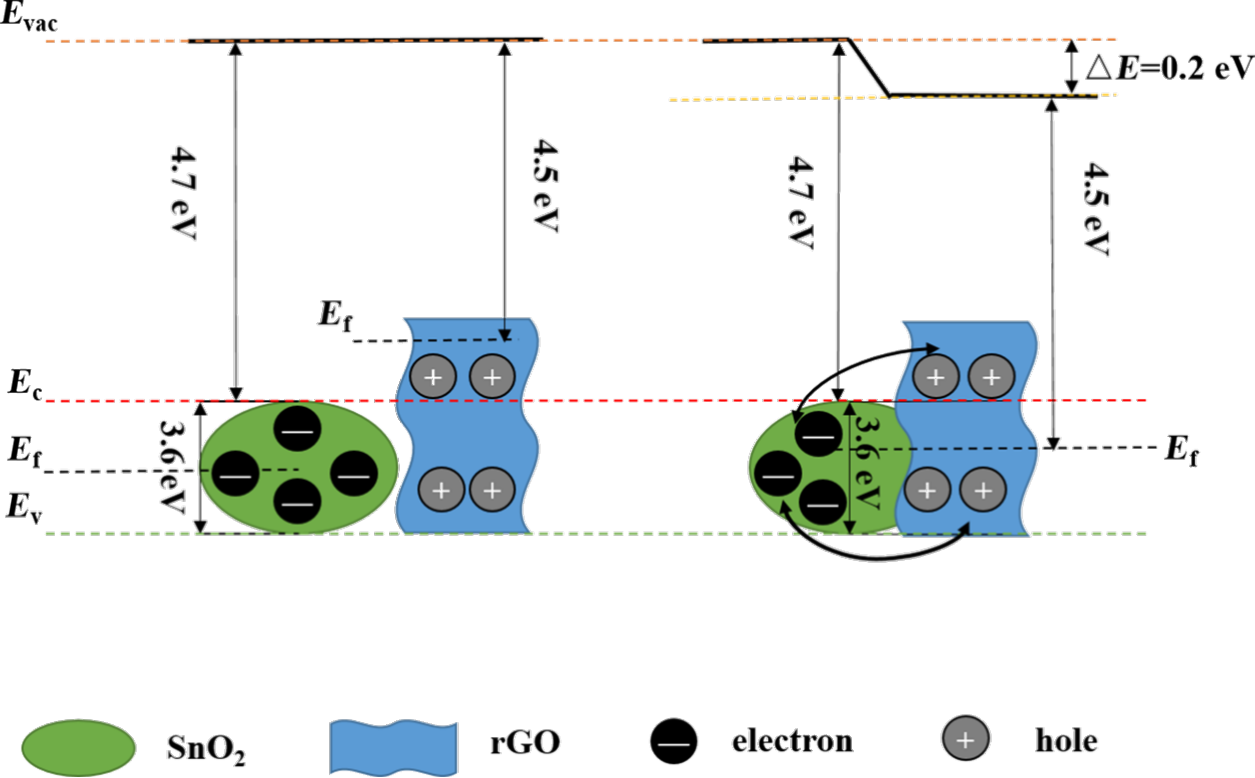
The band diagram of SnO_2_–rGO before and after the combination.

Reduced graphene oxide (rGO), which plays the role of a p-type semiconductor, can form heterojunctions when forming composites with most metal-oxide semiconductors. In the example of a SnO_2_–rGO sensor [45], SnO_2_ and rGO formed p–n heterojunctions. The enhancement mechanism of the p–n heterojunction is shown in Figure 1, *E*_c_ and *E*_v_ are the energies of conduction band and valence band of the two components, respectively, while the Fermi level energy (*E*_f_) is between these two bands. It can be seen from Figure 1 that the work function of rGO is lower than that of SnO_2_, meaning that electrons transfer from rGO to SnO_2_ in the heterojunctions. The Schottky barrier is only 0.2 eV due to the changed Fermi level of the composite structure after achieving a dynamic balance of the electron flow, indicating that the electrons are able to pass through the energy barrier. In summary, the SnO_2_–rGO sensor allow for the transition of electrons even at room temperature because of its low Schottky barrier. When exposed to air at room temperature, oxygen molecules obtain electrons from n-type SnO_2_–rGO hybrids to form O_2_^−^. The electron depletion layers generated on the interfaces of SnO_2_ grains and rGO sheets owing to the loss of electrons and the resistance is the initial resistance of the sensor. The initial resistance of SnO_2_-rGO sensor is much lower than that of a SnO_2_ sensor since because of the high conductivity of rGO, indicating that variations of resistance can be detected at room temperature. Moreover, the electron depletion layers in the SnO_2_–rGO sensor where the electrons are constantly moving between SnO_2_ and rGO are wider than those in a SnO_2_ sensor. When exposed to reducing gases (CO, NH_3_, most VOCs), the gas molecules act as electron donors to SnO_2_–rGO so that the resistance of composite sensor decreases dramatically, leading to high sensitivity and rapid response.

Isotypical p–p homojunctions are formed when rGO forms composites with most p-type metal-oxide semiconductors. The enhancement mechanism of p–p homojunctions is similar to that of p–n heterojunctions. However, p–n junctions cause the recombination of charge carriers with a decreased amount of charge carriers, while p–p junctions lead to spatially separated charge carriers with an unchanged amount of charge carriers. The following summarizes the parameters of several room-temperature gas sensors based on rGO/metal oxides, which exhibit enhanced sensing performance mainly due to the formation of heterojunctions.

Tai et al. [46] deposited ZnO nanoparticles and GO thin films on gold interdigital electrodes (IDEs) through a simple spray process and thermally reduced the deposits to ZnO–rGO composites. The ZnO–rGO sensor exhibited a response of 1.2 to NH_3_ with ultra-fast response/recovery times of 78 s/188 s, which was much better than that of a pure rGO sensor (low response and endless recovery time). The composite sensor with the optimal amount of GO (1.5 mL) was highly sensitive to low concentrations of NH_3_ and responded dramatically, which could be ascribed to the p–n heterojunctions formed between ZnO and rGO. However, the influence of humidity on the sensor response was not negligible due to the residual oxygen (high hydrophilicity) on rGO and the active sites (oxygen vacancies) on ZnO. Chen and co-workers [45] demonstrated that a SnO_2_–rGO sensor, which was synthesized via growing SnO_2_ nanorods on a GO surface, showed a response of 1.3 to 200 ppm NH_3_ with instant response/recovery times of only 8 s/13 s at room temperature. The rapid response and particularly the ultra-fast recovery were extremely inspiring because the recovery time of sensors based on rGO is usually long at room temperature. As a comparison, the pure SnO_2_ sensor is only able to work at 160 °C. The outstanding sensing performance was accounted for by the p–n heterojunctions according to the explanation of the authors. NH_3_ adsorbed on rGO has a smaller adsorption energy than other gases. Strong hydrogen bonds were formed between hydrogen atoms (NH_3_) and the residual oxygen atoms on rGO, facilitating the interaction of NH_3_ with rGO. Thus the selectivity to NH_3_ was good. The authors found that water molecules affected the sensor at low humidity levels. However, the response of the composite sensor increased with increasing humidity at high humidity levels. At low humidity levels, ammonia and water molecules competed for adsorption, which reduces the sensing performance of NH_3_. As humidity levels increased, ammonia adsorbed on the surface of sensor by dissolving into water, leading to the higher response. This is quite different from the usual opinion that sensitivity will decrease at high humidity levels.

Zhang et al. [47] synthesized CuO nanoflowers via hydrothermal method, then CuO and rGO were deposited on the substrate with Ni/Cu IDEs to fabricate the CuO–rGO sensor. The CuO–rGO sensor showed a three-times higher sensitivity to CO and faster response/recovery time than the rGO sensor, while the pure CuO sensor showed no response to CO at room temperature. The p–p junctions constituted between p-type CuO and rGO contributed to the extraordinary sensing performance. The work function of CuO (4.1–4.3 eV) and rGO (5.0–5.1 eV) are not equal [48–49], hence *E*_g1_ (1.2 eV) is not equal to *E*_g2_ (0.4 eV) [50–51]. The electrons will transfer from CuO to rGO until the Fermi energy level of the two components is equal, which accounts for the improved sensing performance to CO at room temperature. Wang et al. [52] mixed ZnO nanowires, prepared via carbothermal reduction, with GO under a protective gas atmosphere (Ar) at 300 °C to synthesize ZnO–rGO hybrids. Although the response to NH_3_ was not as well as that of a pure ZnO sensor, the ZnO–rGO sensor exhibited rapid response/recovery times with a detection limit of 50 ppb while the pure ZnO sensor was unrecoverable at room temperature. The authors stated that the p–p junctions constituted ZnO and rGO reduced the response/recovery times dramatically. An interesting phenomenon found by the authors was that the pure ZnO sensor showed characteristics of a p-type semiconductor during the test. Two reasons were given for this. One was that a little carbon and nitrogen might be doped into ZnO due to the addition of graphite and nitrogen gas protection during the carbothermal reduction (1150 °C). Another was the formation of Schottky barriers between ZnO and the metal electrodes, which caused ZnO to exhibit p-type semiconductor properties. The gas-sensing performance parameters of the abovementioned sensors for reducing gases based on metal oxides and rGO enhanced by the formation of semiconductor interfaces are listed in Table 1.

**Table 1:** Gas-sensing performance of graphene/metal-oxides sensors for reducing gases at room temperature.

target gas	sensor material	synthesis method	conc. (ppm)	response	τ_res_/τ_recov_ (s)	ref.

NH_3_	SnO_2_ nanorods–rGO	hydrothermal	200	1.3	8/13	[45]
NH_3_	SnO_2_–Pd–rGO	one-pot route	5	7.6%	420/3000	[53]
NH_3_	ZnO–rGO	precipitation	10	1.2	78/188	[46]
NH_3_	ZnO nanowires–rGO	thermal reduction	50	19.2%	50/250	[52]
NH_3_	ZnO–rGO	hydrothermal	1	24%	180/150	[54]
CO	CuO–rGO	LBL self-assembly	1	2.56%	70/160	[47]

**Figure 2:**
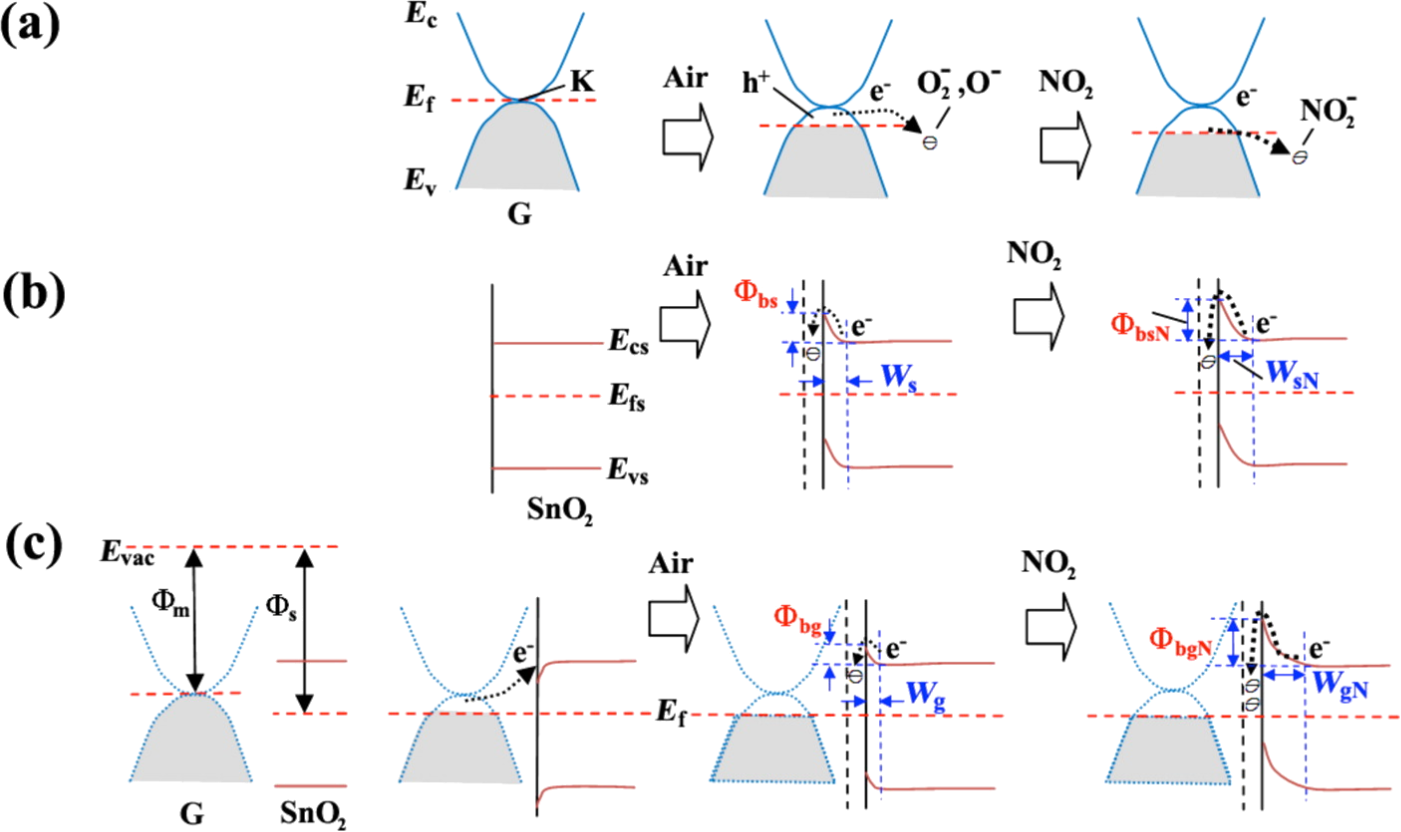
An illustration of the formation of p–n heterojunctions in SnO_2_–rGO hybrids. Reproduced with permission from [56], copyright 2015 American Chemical Society.

Similarly, taking a SnO_2_–rGO sensor as an example [55], as displayed in Figure 2 [56], SnO_2_ and rGO formed p–n heterojunctions during the recombination process. The Fermi level of SnO_2_ is higher than that of rGO since the work function of SnO_2_ (4.55 eV) is lower than that of rGO (4.75 eV). As a result, electrons transfer from SnO_2_ to the conduction band of rGO, not only leading to the bending of the energy bands but also forming potential barriers at the interfaces between SnO_2_ and rGO. When exposed to air at room temperature, oxygen molecules form O_2_^−^ by obtaining electrons from p-type SnO_2_–rGO hybrids. Thus, hole accumulation layers and potential barriers are generated at the interfaces of SnO_2_–rGO hybrids. There are wider hole accumulation layers and higher potential barriers in SnO_2_–rGO than in pristine SnO_2_ owing to the existence of the immanent potential barrier and the imprisoned electrons in rGO. When exposed to an oxidizing gas (NO_2_), which has a high electron affinity, the electrons are continuously captured from SnO_2_–rGO hybrids, leading to wider hole accumulation layers and increased potential barriers. Consequently, the resistance of composite sensor decreased sharply, leading to high sensitivity and rapid response time. The reaction equations are as follows:

[5]
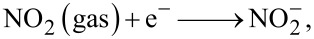


[6]
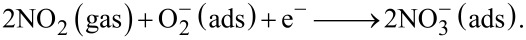


In an early work, Liu et al. [57] stated that a ZnO nanowalls–rGO sensor, which was prepared by growing ZnO nanowalls on rGO films, reached a response of 9.61 to 50 ppm NO_2_ and the response/recovery times were only 25 s/15 s with good stability at room temperature. As a comparison, the response of the ZnO sensor was 6.2 and the response time was over 150 s under the same conditions. The pure rGO sensor only showed a response of 1.2 and a recovery time up to 58 s. The effect of humidity on this sensor was also tested. It was found that the sensor was relatively stable to different humidity levels. The authors ascribed the enhanced sensing properties to both the p–n heterojunctions and the increased carrier concentrations. Specifically, the addition of rGO increased carrier concentrations and provided conductive pathways, which favored the transfer of electrons. The CeO_2_–rGO sensor reported by Jiang et al. [58] exhibited 8.2-times higher sensitivity and faster response than that of a pure rGO sensor at room temperature. The p–n heterojunctions are responsible for the exceptional NO_2_ sensing performance of CeO_2_–rGO sensor. Zhang et al. [59] added GO suspension to a Fe(NO_3_)_3_·9H_2_O solution, then synthesized α-Fe_2_O_3_–rGO hybrids via hydrothermal method. During the process of testing, the authors found that the doping amount of graphene significantly affected the sensing properties of the α-Fe_2_O_3_–rGO sensor. The α-Fe_2_O_3_–rGO sensor with the optimal doping amount of 12.2% showed the highest sensitivity and rapid response time to NO_2_ at room temperature. In addition, the sensor exhibited excellent selectivity to NO_2_ because other interference gases required high operating temperatures to react with the surface of this sensor. As a comparison, the pure α-Fe_2_O_3_ sensor does not work at room temperature. The formation of p–n heterojunctions was an important factor for the exceptional sensing performance. There were two different depletion layers and potential barriers in the composites due to the heterojunctions, one at the α-Fe_2_O_3_ grain boundaries and another at the interfaces of α-Fe_2_O_3_ and rGO. It appeared clear that O_2_ adsorption on the α-Fe_2_O_3_ grain boundaries modified the width of the α-Fe_2_O_3_ depletion layer, which conversely altered the width of depletion layer at the α-Fe_2_O_3_ and rGO interfaces leading to higher sensitivity. In addition, the periodic exposure of the α-Fe_2_O_3_–rGO sensor to 0.1 ppm NO_2_ indicated that the process of response was repeatable. However, a drift of the baseline was easily noted, which usually appears in room-temperature sensors, and further optimization is needed to control the drift. In another work, p–p junctions were accounted for the outstanding sensitivity to NO_2_ of Co_3_O_4_–rGO sensors at room temperature [60].

Liu et al. [61] prepared sulfonated reduced graphene oxide (S-rGO) via adding a solution of diazonium salt into a dispersion of partially reduced GO, then SnO_2_ nanoparticles were grown on S-rGO sheets to prepare SnO_2_–S-rGO hybrids. The SnO_2_–S-rGO sensor exhibited exceptional sensitivity to NO_2_ with a detection limit of 450 ppb at room temperature. The sensor also showed good repeatability and was not affected by water molecules. In contrast, a sensor based on S-rGO showed high sensitivity to NO_2_, but its response/recovery times were long (more than a few minutes). A gas sensor based on SnO_2_–rGO [62] also exhibited excellent response to NO_2_ in their previous study, but it could only work at 50–55 °C. The authors ascribed the better sensing performance of the SnO_2_–S–rGO sensor to the following factors: The addition of sulfonic acid enhances the dispersibility of rGO. At same time, the conductivity of S-rGO is better than that of rGO. Most importantly, the p–n heterojunctions between S-rGO and SnO_2_ lead to the good sensing properties. Because noble metals offer exceptional catalytic activity, the same group [55] developed Ag–SnO_2_–rGO ternary hybrids by reducing AgNO_3_ on the dispersion of SnO_2_–rGO, and the sensing properties were tested at room temperature. The authors demonstrated that the response and recovery of this sensor were much faster than that of SnO_2_–rGO sensor, which could only work at 50–55 °C. The doping of Ag nanoparticles not only improved the electron transfer rate of the sensor, but also increased the number of active sites on the surface of the sensor. Moreover, the introduction of Ag nanoparticles reduced the Schottky barrier of the ternary composites so that those electrons with lower energy were able to cross the energy barrier at room temperature. Thus the sensor can work near room temperature. The most important reason for the excellent NO_2_ sensing performance of the ternary composite sensor was still the p–n heterojunctions. The gas-sensing performance parameters of the abovementioned NO_2_ sensors based on metal oxides and rGO enhanced by the formation of semiconductor interfaces are listed in Table 2.

**Table 2:** NO_2_ sensing performance of graphene/metal-oxide sensors at room temperature.

sensor material	synthesis method	conc. (ppm)	response	τ_res_/τ_recov_ (s)	ref.

Ag–rGO-SnO_2_	hydrothermal	5	2.17	49/339	[55]
ZnO nanowalls–rGO	soft solution	50	9.61	25/15	[57]
CeO_2_–rGO	spray	10	20.5%	92/-	[58]
α-Fe_2_O_3_–rGO	hydrothermal	5	8.2	126/2400	[59]
Co_3_O_4_–rGO	hydrothermal	5	26.8%	90/2400	[60]
SnO_2_–S-rGO	hydrothermal	5	12.03	40/357	[61]
ZnO–rGO	solvothermal	5	25.6%	165/499	[63]
WO_3_–Fe-rGO	precipitation	3	5.9%	1500/7200	[64]
flower-like In_2_O_3_–rGO	hydrothermal	1	1098	—/—	[65]

### Enhancement by improved morphology and structure

**Figure 3:**
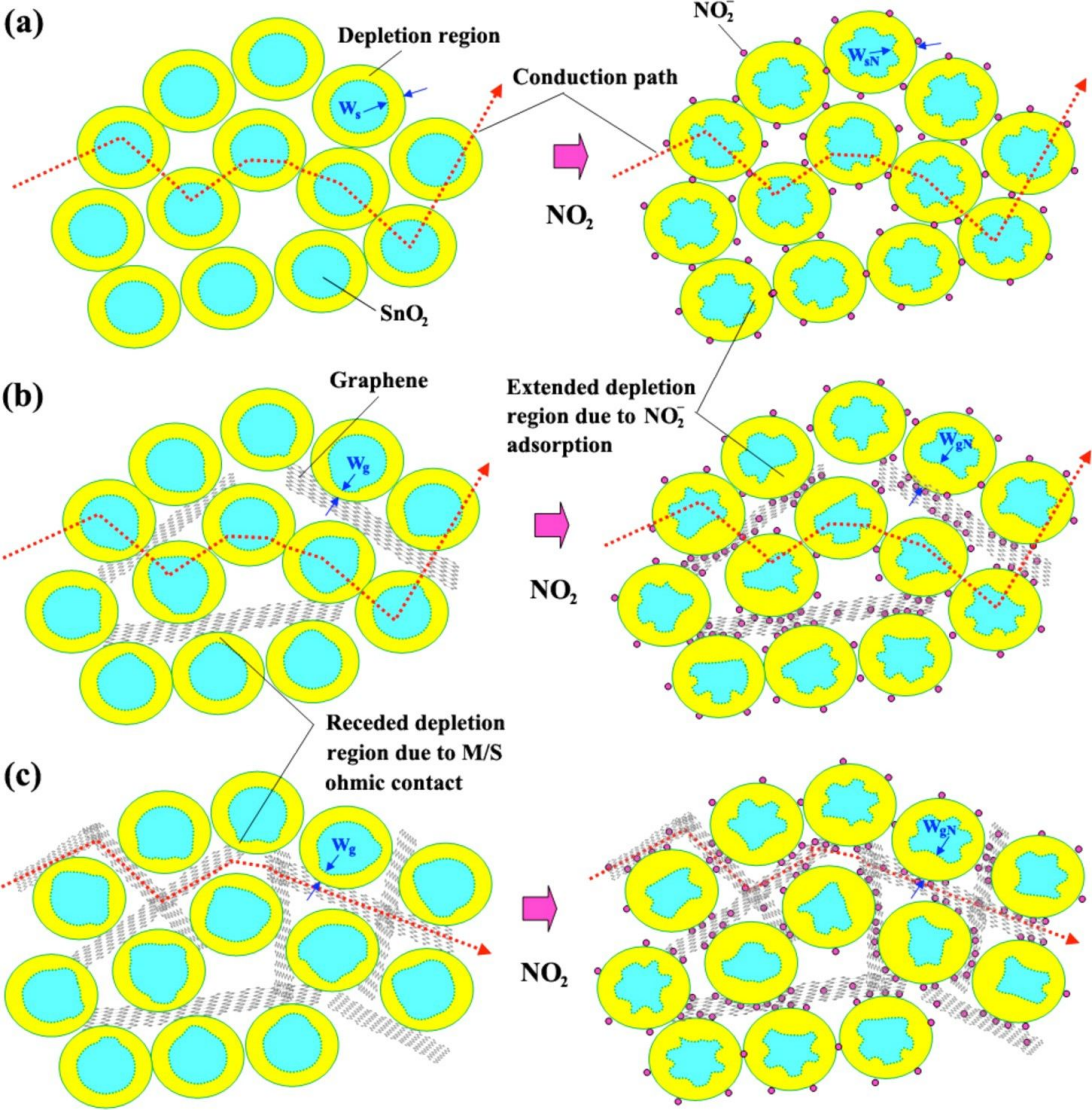
Representative physical models for NO_2_ sensing mechanisms of (a) SnO_2_ nanoparticles and SnO_2_ nanoparticles loaded with graphene at (b) moderately low and (c) high graphene concentrations. Reproduced with permission from [56], copyright 2015 American Chemical Society.

By changing morphology and structure of the sensor materials, one can obtain large specific surface area, more conductive pathways and more active sites, which significantly improve sensing performance of the sensor. As shown in Figure 3 [56], SnO_2_ nanoparticles prevent graphene from agglomerating, which in turn leads to a high specific surface area. Graphene enhances the conductivity of the composite materials, enabling the composite sensors to achieve a high response at low operating temperatures. Moreover, the introduction of graphene provides more adsorption sites at the surface of the composite so that the response can be significantly improved.

ZnO is widely used as a typical wide-bandgap (3.37 eV) metal-oxide gas sensor material. However, the problem with ZnO gas sensors is their poor selectivity [66]. Li et al. [67] synthesized urchin-like ZnO nanorods–graphene via a facile solvothermal method. The urchin-like morphology of the samples provided a large specific surface area. The response of the sensor was 17.4% to 100 ppm NO_2_ at room temperature, while the pure rGO sensor only exhibited 7.7% response under the same conditions. Apart from the conductive 3D network channels provided by rGO, the large specific surface area of the composite sensor also contributed to the high response. Liu et al. [68] developed a 3D ZnO–rGO aerogel by heating (200 °C for 10 h) a mixture of ZnCl_2_, GO, sodium acetate and sodium citrate in an autoclave. Different from other drying processes, the hybrids were obtained through freeze-drying to keep the 3D structure. ZnO nanoparticles were well wrapped in graphene sheets, while the graphene sheets were well dispersed in the hybrids. The ZnO–rGO sensor exhibited 8% response to 50 ppm NO_2_ with rather rapid response/recovery times (132 s/164 s) while the pure rGO sensor showed a response of 6.4% with longer response/recovery times (149 s/243 s) at room temperature. The sensor showed exceptional selectivity to NO_2_. Other gases, such as C_3_H_6_O, CH_3_(CH_2_)_3_OH, CH_3_OH and H_2_, were not able to react with the oxygen ions that adsorbed on the surface of this composite sensor at low operating temperatures. NO_2_ is a strongly electron-withdrawing molecule that enhanced the electron-withdrawing ability of oxygen functional groups through its electron-deficient N atom interacting with active sites on rGO. In addition, the authors did an interesting comparative experiment with the ZnO–rGO sensor, a pure rGO sensor and a sensor fabricated by physically mixing ZnO and graphene dispersions. The last sensor exhibited the worst gas-sensing performance to NO_2_ due to the agglomeration of graphene sheets and ZnO particles. Similarly, this group [69] demonstrated that a room-temperature sensor composed of a 3D graphene aerogel and SnO_2_ nanoparticles, synthesized via the method mentioned above, exhibited a higher response to NO_2_ and faster response/recovery times than a 2D SnO_2_–graphene sensor, fabricated by the same method without freeze-drying process.

Titanium dioxide (TiO_2_), as a wide-bandgap semiconductor, has been widely used as photocatalyst, and in solar cells and gas sensors [70–72]. In general, its operating temperature is over 200 °C, so scholars try to prepare composites with graphene to reduce its operating temperature. However, the stability of this type of composite sensors is a problem. Recently Li et al. [73] reported an ultrafast and sensitive NH_3_ sensor using rGO decorated with TiO_2_ nanocrystals. There were two different morphologies in these sensing materials: rGO either laid on the surface of TiO_2_ nanoparticles, partly wrapping them, or made “bridges” at the interface between two nanoparticles. Due to the “bridges” existing between TiO_2_ nanoparticles, the initial resistance of TiO_2_–rGO sensor was greatly reduced, indicating that the sensor was able to work at room temperature. The partly “wrapping” microstructure enhanced the number of adsorption sites. Moreover, the TiO_2_–rGO sensor showed a better selectivity to NH_3_ than the pure rGO sensor owing to the acidic surface of TiO_2_ preferentially adsorbing primarily NH_3_. Ye et al. [74] stated that a TiO_2_–rGO sensor, fabricated via depositing GO and TiO_2_ nanoparticles on IDEs and then heating them for reduction, showed a 1.5-times higher response than a rGO sensor at room temperature. In contrast, a pure TiO_2_ sensor did not respond to NH_3_ at room temperature, which proved that the introduction of graphene reduced the operating temperature of the TiO_2_ sensor. The same group [75] reported that a NH_3_ room-temperature sensor based on TiO_2_–rGO hybrids, synthesized by a hydrothermal method, exhibited a two-times higher response and a much shorter response time than the TiO_2_–rGO sensor fabricated by direct deposition as mentioned above. Apart from the porous and undulating graphene sheets due to the introduction of TiO_2_ nanoparticles, the mellow and regular TiO_2_ nanoparticles also contributed to the improvement of the gas-sensing performance. However, the stability was poor because the composite sensor was sensitive to water molecules. Further optimization is needed to control the influence of humidity on the sensors.

SnO_2_, a semiconductor with a bandgap of 3.62 eV, has an exceptional response to toxic industrial gases. However, a pure SnO_2_ sensor has low sensitivity at low concentrations of gases [76–77], so the combination of graphene and SnO_2_ has attracted widespread attention. In a pioneering work, Lin et al. [78] demonstrated that SnO_2_–graphene (GN) hybrids fabricated via hydrothermal synthesis using GO and SnCl_2_ as precursors exhibited a 3D nanostructure with high specific surface area (94.9 m^2^/g). During the hydrothermal process, GO served as a template to promote the preferential growth of SnO_2_ nanoparticles and prevented SnO_2_ nanoparticles from agglomeration. The response of the composite sensor to NH_3_ at 10 ppm was 5.09% and the response/recovery time was less than 1 min at room temperature, whereas the sensor based on SnO_2_ did not respond to NH_3_ and the sensor based on GN showed a response of only 2.7 % at room temperature. The authors claimed that the introduction of GN not only increased the specific surface area, but also improved the conductivity of the sensor at room temperature. Bo et al. [79] grew vertical graphene (VG) on the surface of Pt IDEs, then used chronoamperometry to deposit SnO_2_ nanoparticles on the VG networks. The SnO_2_–VG room-temperature sensor was capable of detecting as low as 20 ppb of formaldehyde and showed a response of 4.6% to 5 ppm formaldehyde, which was three-times higher than that of the graphene sensor. Compared with normal graphene sheets, the specific surface area of vertical graphene sheets is extremely increased. The SnO_2_ nanoparticles on the VG sheets with the 3D structure provided numerous adsorption sites for target gas molecules.

**Figure 4:**
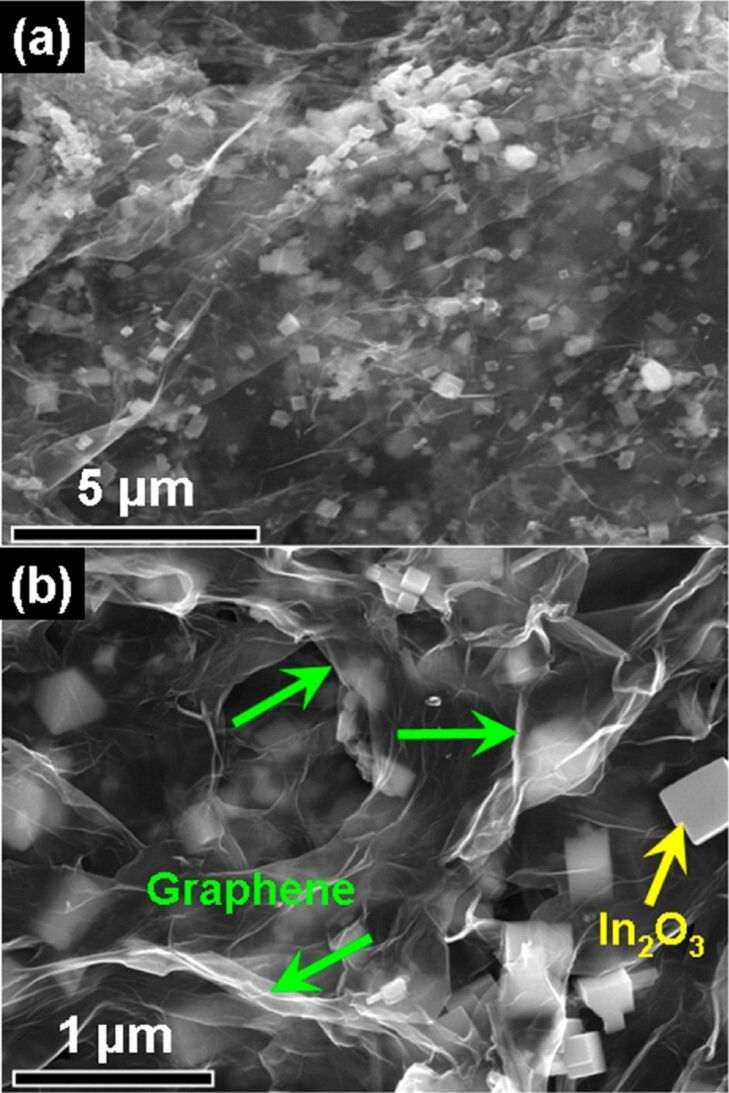
SEM images of the In_2_O_3_ cubes-rGO composites at different magnification. Reproduced with permission from [80], copyright 2014 American Chemical Society.

Apart from the abovementioned common MOS, some other MOS also have exceptional sensing properties after mixing with graphene. Yang et al. [80] added a GO suspension, prepared via a modified Hummers method, to a solution of In(NO_3_)_3_ to develop In_2_O_3_–rGO hybrids through a facile one-step microwave-assisted hydrothermal method. The response of the In_2_O_3_–rGO sensor to 5 ppm NO_2_ was 37.81% with excellent stability and selectivity at room temperature. It should be noted that graphene improved the conductivity of the sensing materials, while the addition of In_2_O_3_ nanocubes prevented rGO sheets from re-accumulation, as shown in Figure 4, leading to an increased specific surface areas and a higher number of active sites. Meng and co-workers [81] published an inspiring study, where they develop a microwave-assisted hydrothermal technique to grow CuO rods in GO suspension using cetyltrimethylammonium bromide (CTAB) as a soft template. The Cu_2_O nanorods–rGO hybrids obtained after annealing showed a porous structure with a high surface area to volume ratio. The porous structure promoted the diffusion of gases, improving the reaction of gases with oxygen species on the surface of the hybrid material. The Cu_2_O–rGO composites exhibited exceptional catalytic activity and acted as high-efficiency catalysts for the reduction of oxygen molecules, leading to an excellent response. This room-temperature sensor exhibited a linear response to the concentration of NH_3_ with rapid response/recovery times. The gas-sensing performance parameters of the abovementioned graphene/metal-oxide gas sensors enhanced by improved morphology and structure are listed in Table 3.

**Table 3:** Gas-sensing performance of graphene/metal-oxide sensors at room temperature.

target gas	sensor material	synthesis method	conc. (ppm)	response	τ_res_/τ_recov_ (s)	ref.

NO_2_	ZnO–rGO	solvothermal	100	17.4%	780/1980	[67]
NO_2_	ZnO–rGO aerogel	solvothermal	50	8%	132/164	[68]
HCHO	ZnO–rGO	hydrothermal	2	2%	60/—	[82]
HCHO	ZnO–rGO	CVD	9	52%	36/—	[83]
NH_3_	TiO_2_–rGO	hydrothermal	30	3.3%	—/—	[73]
NH_3_	TiO_2_–rGO	precipitation	10	0.62	55/200	[74]
NH_3_	TiO_2_–rGO	hydrothermal	10	1.7	114/304	[75]
NO_2_	SnO_2_–rGO aerogel	solvothermal	50	6%	190/224	[69]
NH_3_	SnO_2_–GN	hydrothermal	10	5.9%	<60/<60	[78]
HCHO	SnO_2_–VG	CVD	5	4.6%	46/95	[79]
NO_2_	In_2_O_3_–rGO	hydrothermal	5	37.81%	—/—	[80]
NO_2_	In_2_O_3_–rGO	hydrothermal	30	8.25	240/1440	[85]
NH_3_	Cu_2_O–rGO	hydrothermal	200	2.04	28/206	[81]
NO_2_	Cu*_x_*O–graphene	vacuum-assisted reflux	0.097	27.1%	58.7/—	[87]
NO_2_	WO_3_–rGO	one-pot polyol	5	769%	540/1080	[84]
NO_2_	α-Fe_2_O_3_–rGO	hydrothermal	90	150.63%	—/1648	[86]

### Enhancement by a synergetic coupling effect between graphene and metal oxides

In the past years, some scholars have found that excellent sensing properties can also be achieved when MOS are directly mixed with pristine graphene. The formation of heterojunctions is improper to explain this phenomenon since pristine graphene is a conducting material. Also, the morphology and structure of the composite sensor have not been altered through the introduction of graphene. Another strengthening mechanism, namely a synergetic coupling effect between graphene and metal oxides, is proposed. In detail, the enhanced sensing performance is accounted for by chemical bonds between graphene and metal oxides. Many XPS studies have claimed that there indeed exist chemical bonds between metal oxides and graphene.

**Figure 5:**
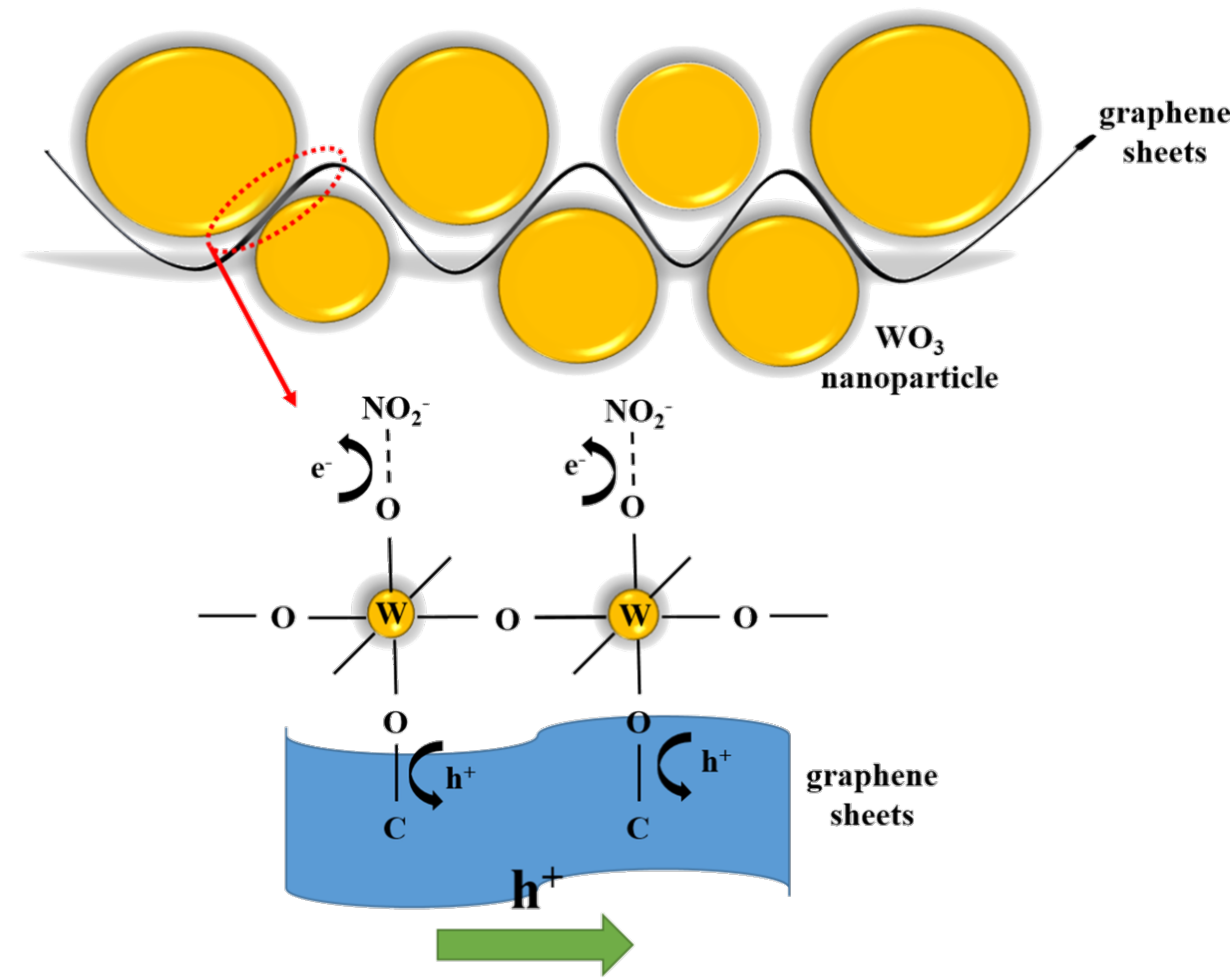
Proposed NO_2_-sensing mechanism of GR–WO_3_ composites at room temperature and electron transfer between WO_3_ nanospheres and graphene sheets.

WO_3_, a transition-metal oxide semiconductor is widely used as a gas sensor because of its small bandgap (2.585 eV) and its physical and chemical stability [88–89]. In an early work, Jie et al. [90] reported that a NO_2_ sensor based on WO_3_ nanospheres wrapped in graphene sheets, prepared by a simple sol–gel technique, showed a linear response to low concentrations of NO_2_, while the pure WO_3_ and graphene sensors did not respond to NO_2_ at room temperature. The authors claimed that the reason for room-temperature sensing of the composite sensor was the effective charge transfer between graphene and WO_3_ nanospheres by chemical bonds. The research group confirmed that there existed C–O–W chemical bonds between WO_3_ and graphene by Raman and XPS measurements. The proposed sensing mechanism is shown in Figure 5. When exposed to oxygen or NO_2_ molecules, the gas molecules adsorbed on WO_3_ nanospheres cause the energy band to bend upward via obtaining electrons from WO_3_ and move the Fermi level of WO_3_ from the conduction band to the valence band. The reactions mentioned above shift the work function of WO_3_ [91] to be adjacent to graphene, leading to the electrons moving easily at the interfaces of WO_3_ and graphene. Because of the continuous loss of electrons of WO_3_, electrons are transferred from the graphene sheets to WO_3_ through chemical bonds to maintain the adsorption. The chemical bonds are considered as electrons bridges during the response, which improved the sensing performance of the graphene–WO_3_ sensor. In addition, the excellent conductivity of graphene may also enhance the response because graphene offers conductive pathways that enhanced the efficiency of charge-carrier transfer in the composites. Zhang et al. [60] fabricated a Co_3_O_4_–graphene gas sensor through a traditional hydrothermal method. The XPS results certified that Co–O–C bonds were formed at the interfaces of Co_3_O_4_ and graphene. The Co cations in Co_3_O_4_ were strongly attracted by the oxygen anions in the oxygen functional groups of the graphene. Consequently, the centers of Co^3+^ and Co^2+^ served as additional active adsorption sites for NO_2_. At the same time, the electrons transferred from graphene to Co_3_O_4_ through the Co–O–C bonds lead to an additional increase of the width of hole accumulation layers, leading to a high sensitivity at room temperature. In another work, Feng et al. [92] developed composite nanofibers of rGO-coated Co_3_O_4_ nanocrystals by using electrospinning. The Co_3_O_4_–rGO room-temperature sensor showed excellent sensitivity to low concentrations of NH_3_, an ultrafast response time of only 4 s with exceptional selectivity to NH_3_. The authors claimed that the introduction of rGO exhibiting a strong attraction to NH_3_ played a crucial role in improving the sensitivity and selectivity. However, the most important factor was that the chemical bonds (C–O–Co) formed between rGO and Co_3_O_4_, which facilitated the electrons transfer at the interfaces of rGO and Co_3_O_4_.

Kumar and co-workers [93] fabricated SnO–graphene gas sensors through heating mixtures of SnCl_2_ and graphene. The response of the SnO–graphene sensor to 50 ppm NH_3_ reached 21% with fast response/recovery times of only 15 s/30 s at room temperature. The most encouraging result was that the sensor could maintain its excellent response for more than one year. The authors demonstrated that the formation of C–O–Sn chemical bonds, confirmed by XPS results, was responsible for the superior performance of the SnO–graphene sensor. Zhang et al. [94] fabricated a room-temperature NiO–graphene sensor that exhibited a better sensitivity to NO_2_ than a pure NiO sensor. The authors also ascribed the enhanced sensor sensitivity to the Ni–O–C bonds formed at the interfaces of NiO nanoparticles and graphene sheets. The NO_2_ molecules capture electrons from the surface of NiO nanoparticles when the sensor is exposed to NO_2_. More and more electrons are transferred from graphene to NiO by Ni–O–C bonds because of the lower Fermi level of NiO, increasing the sensitivity and response rate of the NiO–graphene sensor. However, the sensitivity of the NiO–graphene sensor was not as good as that of the pure NiO sensor at low concentrations of NO_2_ (0.25–1 ppm). In order to enhance the sensitivity of this sensor, the authors [95] added n-type semiconductor SnO_2_ to the NiO–graphene hybrids (p-type) to form p–n heterojunctions and found that the sensitivity of the SnO_2_–NiO–graphene sensor was about 10-times that of the NiO–graphene sensor. The authors claimed that the low adsorption energy of NO_2_ on the surface of SnO_2_ and the formation of p–n heterojunctions contributed to the exceptional sensitivity. The gas-sensing performance parameters of the abovementioned metal oxide–graphene gas sensors enhanced by a synergetic coupling effect are listed in Table 4.

**Table 4:** Gas-sensing performance of graphene/metal-oxide sensors at room temperature.

target gas	sensor material	synthesis method	conc. (ppm)	response	τ_res_/τ_recov_ (s)	ref.

NO_2_	Co_3_O_4_–rGO	hydrothermal	5	26.8%	90/2400	[60]
NO_2_	Co_3_O_4_–rGO	hydrothermal	60	82%	300/—	[101]
NH_3_	Co_3_O_4_–rGO	electrospinning	5	53.6%	4/300	[92]
NO_2_	NiO–rGO	solvothermal	60	6.2	—/—	[94]
NH_3_	SnO–graphene	CVD	50	21%	15/30	[93]
NO_2_	SnO_2_–NiO–rGO	hydrothermal	60	62.27	220/835	[95]
NO_2_	SnO_2_–graphene	sol–gel	20	9.6%	60/300	[100]
NH_3_	TiO_2_–Pd–rGO	one-pot polyol	10	14.9%	184/81	[96]
HCHO	TiO_2_–rGO	thermal reduction	0.5	0.4	70/126	[97]
NH_3_	ZnO–rGO	precipitation	0.5	5.6	6/36	[98]
HCHO	ZnO–rGO	solution-processed	25	0.43	30/40	[99]
NO_2_	WO_3_–GR	sol–gel	7	11.6%	—/—	[90]

## Summary and Outlook

In this review article, the progress of room-temperature graphene/metal-oxide gas sensors has been summarized. The introduction of graphene or its derivatives greatly improves the sensitivity and reduces the operating temperature of MOS gas sensors. The advances in the field of graphene/metal-oxide gas sensors over the past few years have greatly broadened the research directions of gas sensors. However, the achievements reported in this article are still in the stage of basic research and require a lot of effort to put these researches into practical use. The main bottleneck is that the gas sensors are susceptible to environmental humidity [45–47,52,73–75,83,87,96]. In a recent study, a humidity sensor based on SnO_2_–rGO fabricated by Zhang et al. [102] showed a high sensitivity over the full range of humidity at room temperature, which demonstrated that these types of composite sensors are moisture-sensitive by nature. Although several researches demonstrate that the properties of these sensors are capable to be maintained for more than half a year, such stability has been only obtained in laboratory environments, indicating that it is difficult to have such long-term stability in the real environment. Another problem is to explore the ability to detect nonpolar and large molecules, such as volatile organic compounds (VOCs) which are extremely harmful to human health and the environment. The interaction of these molecules with graphene/metal-oxide sensors is different from that of the polar molecules, and graphene showed low surface affinity to these molecules, which makes detection difficult.

A small number of these composite sensors seem to exhibit the phenomenon of resistance-baseline drift, which is due to the formation of chemical bonds during the adsorption of gas molecules at the material surface, resulting in slow or even impossible recovery. Some researchers have stated that the recovery time can be reduced by heating or irradiating with UV light. Unfortunately, the measures will damage the structure of rGO films and increase the cost. Another point we have to mention is that the sensors based on metal oxides and graphene exhibit complicated sensing mechanisms owing to the additional reactions between gas molecules and various sensing materials.

Future works should focus on the following aspects: The most important thing is to simulate the influence of humidity on the sensor performance in a real environment and study the mechanism of humidity influence. A hydrophobic coating can be applied to the surface of the sensor to reduce the effects of humidity. Regarding the synthesis, it is important to quantify and control the effect of hydrothermal or solvothermal parameters on the micromorphology of hybrids. An important way to enhance the selectivity of composite sensors is mixing or doping with other phases (noble metals or conducting polymers). Similarly, the recovery time can be reduced by surface functionalization of graphene/metal-oxide hybrids with specific functional molecules such as boron or nitrogen. In addition, more effort should be dedicated to the detection of more environmental gases, especially VOCs. Last but not least, the influence of baseline drift in resistance can be eliminated by defining a new method, which calculates the response values by using the resistance value after each response cycle.
